# Heterogeneity of Rabies Vaccination Recommendations across Asia

**DOI:** 10.3390/tropicalmed2030023

**Published:** 2017-07-06

**Authors:** Philippe Buchy, Scott Preiss, Ved Singh, Piyali Mukherjee

**Affiliations:** 1 GSK, 23 Rochester Park, Singapore 139234, Singapore; philippe.x.buchy@gsk.com; 2 GSK, 20 Avenue Fleming, 1300 Wavre, Belgium; scott.s.preiss@gsk.com (S.P.); ved.s.singh@gsk.com (V.S.)

**Keywords:** Asia, guidelines, prevention and control, rabies, vaccination

## Abstract

Asian countries bear the greatest burden of the disease, with a majority (59%) of rabies-related deaths occurring in Asia. In order to promote best practices, we summarized national human vaccination guidelines across this region, to highlight differences and similarities and to discuss the aspects that would benefit from updates. National management guidelines for rabies were retrieved from various sources to extract information on rabies pre- and post-exposure prophylaxis (PrEP, and PEP), booster vaccination, and route of administration. Rabies guidelines recommendations for wound management and PrEP across Asia are broadly aligned to the World Health Organization (WHO) guidelines. For PEP, the 5-dose Essen, and the 4-dose Zagreb are the regimens of choice for intramuscular (IM), and the Thai Red Cross regimen for intradermal (ID), administration. Several national guidelines have yet to endorse ID vaccine administration. Most guidelines recommend rabies immunoglobulin in category III exposures. Booster recommendations are not included in all guidelines, with limited clarity on booster requirement across the spectrum of risk of rabies exposure. In conclusion, national recommendations across Asian countries differ and while some guidelines are closely aligned to the WHO recommendations, resource-saving ID administration and use of rational abbreviated schedules have yet to be endorsed.

## Highlights

- The recommended practices for rabies pre- and post-exposure prophylaxis vary widely from country to country in Asia.
- Overall, the WHO recommendations for wound management are consistent in national guidelines. The post-exposure prophylaxis (PEP) 5-dose Essen and 4-dose Zagreb intramuscular (IM) regimens are uniformly recommended.
- The value of intradermal (ID) administration in reducing costs is not considered in several country guidelines.
- In the majority of the national recommendations, concurrent administration of rabies immunoglobulin (RIG) in category III exposures is recommended; however, there are concerns regarding availability and cost.
- Booster recommendations are not included in all guidelines, with limited clarity on booster requirement across the spectrum of risk of rabies exposure.
- Limited recommendations are available for special populations including pregnant women, aged population, and immunocompromised patients.

## 1. Introduction

Despite being entirely preventable, human rabies is estimated to cause 59,000 global deaths annually, of which 59% occur in the Asia region [1,2]. Bites from infected dogs cause 95% of human rabies deaths, 40% of which occur in children [3,4]. If prompt vaccination is not given, rabies infection causes death in virtually all cases [3]. However, the death toll can be considerably reduced through access to post-exposure prophylaxis (PEP), consisting of wound cleaning, rabies immunoglobulin (RIG) and vaccination. Additionally, the broader use of pre-exposure (PrEP) vaccination and mass dog vaccination would further reduce deaths and help control and eventually eliminate dog-mediated human rabies [1,4,5]. Human and canine rabies control are therefore interdependent, and experience has shown that a collaboration between human and animal health sectors is required to enhance cost-effectiveness of rabies control measures, and effectively reduce rabies incidence and associated societal burden [6,7,8]. This approach, under the “One Health” framework, with the goal to eliminate dog-mediated rabies by 2030, is endorsed and jointly advocated by the World Health Organization (WHO), the World Organisation for Animal Health (OIE), the Food and Agriculture Organization of the United Nations (FAO), and the Global Alliance for Rabies Control (GARC) [5].

Activities aiming to control rabies disease have been initiated, and some governments of disease-endemic countries have committed to its elimination by 2030. In this process, it is important to have reliable country-level epidemiology data, active surveillance systems seeking to register any new cases, appropriate vaccine requirement forecasting, and other such tools that can track progress made towards achieving this goal [3].

However, national reporting systems and public awareness are often lacking [2,3]. People living in countries with endemic rabies disease are sometimes insufficiently aware of the risk and the need for vaccination. Furthermore, the total cost of vaccination—including, for instance, travel expenses to-and-from the vaccination center, and lost work income—is a major consideration for these individuals [2,4,9,10,11,12,13]. Moreover, healthcare personnel are sometimes unaware of appropriate wound management, of PEP regimens, and of the existence of PrEP [4,14].

In this context, safe and effective, yet cost-saving and/or shorter regimens are appealing. Both intramuscular (IM) and intradermal (ID) vaccine schedules are endorsed by the WHO (Table 1). ID administration requires fewer vaccine vials than IM, reducing the direct vaccination cost by 60–80% [13]. For optimal cost benefit of the ID administration, the health seeking rate should be sufficiently high to utilize the entire vial within 6–8 h [1,13]. Importantly, patients receiving IM or ID cell culture rabies vaccination should reach 0.5 IU/mL or higher titers of rabies virus neutralizing antibodies (RVNA) within 14 days from vaccination (the level considered indicative of adequate immune response to vaccination). Both vaccines administration methods demonstrate acceptable safety profiles. In this respect, in an otherwise healthy population, ID vaccination is clinically equivalent to that of IM [13]. The shorter, dose-sparing vaccination regimens are equally effective alternatives, improving compliance and allowing for more animal bite victims to successfully complete a full vaccination course [1,13,15,16,17,18,19,20]. However, these have not yet been endorsed by all countries with endemic disease in Asia region. To achieve the 2030 goal for rabies elimination, national PrEP and PEP strategies and clear public health guidelines following WHO recommendations must be implemented to increase access to vaccination with optimal efficacy of the vaccine and control the disease [1,4]. A Working Group on rabies vaccines and rabies immunoglobulins established by the WHO Strategic Advisory Group of Experts on Immunization (SAGE) is currently reviewing new evidence on country practices in the use of RIG, PrEP, and the cost-effectiveness of the interventions. The findings will be discussed and SAGE will consider recommendations on the WHO position on rabies during its October 2017 meeting [21,22].

We collected current national human vaccination recommendations across Asia to summarize and highlight differences and similarities, and to identify best practices. The ultimate aim was to increase knowledge of current needs and identify gaps, in turn encouraging adoption of uniform rabies vaccination methods across all countries to ensure optimization of resource utilization.

## 2. Methods

We aimed to retrieve national guidelines on management of human rabies exposures from 21 Asian countries: Bangladesh, Bhutan, Brunei, Cambodia, China, Hong Kong, India, Indonesia, Japan, Lao People’s Democratic Republic (PDR), Malaysia, Myanmar, Nepal, Pakistan, Philippines, Republic of Korea, Singapore, Sri Lanka, Taiwan, Thailand, and Vietnam. The research for the guidelines was performed between January and March 2017. Our investigation included scientific literature review searches, Ministry of Health web pages, other web pages (e.g., travel information pages), surveillance platforms and databases, medical association guidelines, publications for healthcare professionals and the public, and personal communications with people working on the specific field in each country. English and country-specific websites were reviewed in the local language. Due to the nature of this research, systematic review methods could not be applied. Indeed, national guidelines are not expected to be necessarily published in scientific journals, hence this work did rely mostly on information provided by Ministry of Health websites (often in local language), by national experts, etc. Findings were classified by document type, publisher, and year of publication. We aimed (a) to summarize PEP and PrEP recommendations and compare them to the WHO recommendations; (b) to explore variations between countries in human rabies prophylaxis and discuss opportunities for a harmonized approach; (c) to identify pitfalls and drawbacks in the adaptation of successful human rabies treatment and prophylaxis programs sufficiently harmonized with the WHO recommendations; (d) to highlight best practices.

## 3. Findings

### 3.1. Overall Findings

**Figure 1:**
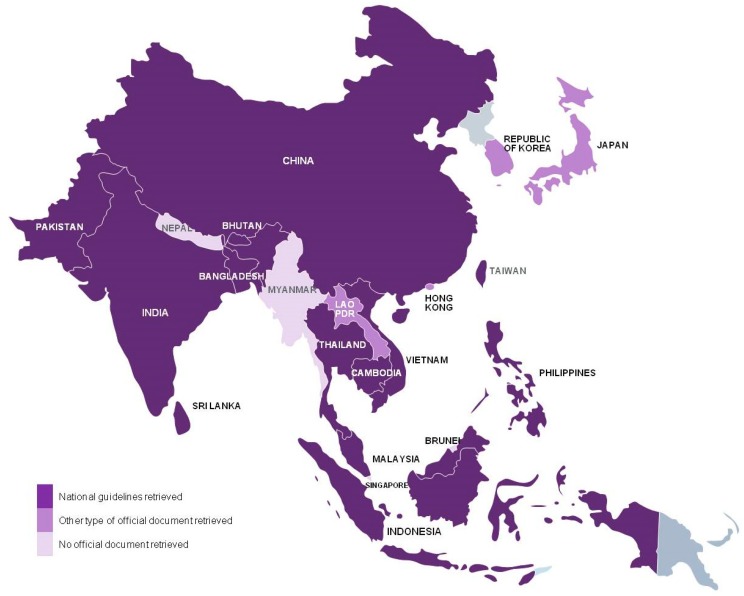
Countries for which national guidelines or other type of rabies-related official document on rabies human vaccination schedules were retrieved and included in the analysis.

We retrieved national vaccination guidelines from 13 of the 21 countries considered: Bangladesh [23], Bhutan [24], Cambodia [25], China [26], India [27,28], Indonesia [29], Malaysia [30], Pakistan [31], Philippines [32], Sri Lanka [33], Taiwan [34], Thailand [35], and Vietnam [36]. Official documents, outlining current rabies prevention and treatment practices, were retrieved for Hong Kong [37,38,39], Japan [40], Lao PDR [41], and the Republic of Korea [42]. The national guidelines retrieved were usually issued by the Ministry of Health. We did not succeed to retrieve national documentation for four countries: Brunei, Myanmar, Nepal, and Singapore (Figure 1 and Table 2).

As expected, all national guidelines were considerably detailed and incorporated the WHO recommendations for PrEP and PEP vaccination. All recommended vaccines were cell culture vaccines and embryonated egg-based vaccines (CCEEVs). The schedules are summarized in Table 2. Overall characteristics are given below.

### 3.2. Post-Exposure Prophylaxis (PEP)

#### 3.2.1. Wound Care

Overall, the WHO recommendations for wound management are consistent in national guidelines. The guidelines from China [26], India [27], and Pakistan [31] were more detailed than others, containing explicit recommendations for wound care with photographs. The Indian [27], Pakistani [31], Philippine [32], and Sri Lankan [33] guidelines recommend avoidance of wound suturing to allow for antibody (RIG) diffusion throughout the tissues, unless there is life-threatening bleeding. The Pakistani guidelines recommend daily dressing instead of suturing, except for very loose suturing for severe facial bites, with proper suturing 2–3 days after initial wound management [31]. Cauterization is no longer recommended in India as it does not offer additional benefit over washing while tetanus and antibiotics should be given if required, and if sepsis prevention is needed [27]. The Philippines recommend adhesive strips as an alternative to suturing, and also include detailed recommendations on antibiotic treatment [32]. In Sri Lanka, wound dressing is recommended, but not suturing [33].

#### 3.2.2. RIG

Most guidelines follow WHO recommendations regarding RIG administration; however, there are some concerns related to the associated cost. Thus, for Lao PDR, the 2000 WHO report shows that RIG is rarely used due to its high cost [41], and in a number of countries [25,32,35], equine immunoglobulin (ERIG) is more commonly used than human immunoglobulin (HRIG) because it is cheaper and therefore more commonly available for free. The Philippine guidelines also contain details on the size of needles, skin tests to check whether human RIG should be preferred, special considerations for bites to the finger and toes, and they recommend HRIG for multiple bites and in symptomatic patients infected with human immunodeficiency virus (HIV) [32].

#### 3.2.3. PEP Vaccination Schedules

- The 5-dose Essen, 4-dose Zagreb, or both schedules were the regimens of choice for IM administration. The Essen is used in Bhutan, Cambodia, China, India, Philippines, Sri Lanka, Taiwan, Thailand, Vietnam; and Zagreb in Bangladesh, Cambodia, China, Indonesia, Pakistan, Philippines, Sri Lanka (Table 2). The 4-dose shortened Essen was the regimen of choice in the Malaysian recommendations, and was also recommended in the guidelines of Philippines (Table 2 and Table 3).
- We found recommendation for ID administration in 9 of the 13 retrieved national guidelines; the guidelines of China, Indonesia, Malaysia, and Taiwan do not include such recommendation (Table 2 and Table 3). Also in the official documents retrieved for Japan and Lao PDR we did not find ID recommendations (Table 2 and Table 3). All guidelines with ID recommendation suggested the updated Thai Red Cross regimen (Table 2 and Table 3).
- The Japanese guidelines recommend only subcutaneous (SC) administration as shown in Table 2.

#### 3.2.4. PEP Vaccination for Re-Exposed Individuals

Not all guidelines advise on the post-exposure management that should be followed when an individual who has previously received rabies vaccination is re-exposed (Table 2). The guidelines of Bangladesh [23], Bhutan [24], China [26], India [27], and Philippines [32] recommend new full vaccination when previous full exposure cannot be documented or is uncertain.

### 3.3. Pre-Exposure Prophylaxis (PrEP)

#### 3.3.1. PrEP Vaccination Schedules

With the exception of Japanese guidelines, all other guidelines recommend IM or ID vaccination schedules (Table 2). IM administration is included in all of the 13 retrieved national guidelines. ID is included in only seven national guidelines, these of Bangladesh, Bhutan, Cambodia, Pakistan, Philippines, Sri Lanka, and Thailand (Table 2). The Malaysian interim guidelines do not include any reference to PrEP [30].

#### 3.3.2. Booster after PrEP Vaccination

Eight national guidelines include booster recommendations and there are differences between countries; all relevant recommendations are described in detail in Table 2. For individuals working under a high risk (laboratory workers dealing with rabies virus and other lyssaviruses) or continuous risk (veterinarians and animal health officers) of exposure to rabies, all recommendations agree that a booster vaccination should be given when antibody titers fall below 0.5 IU/mL.

### 3.4. Vaccination Recommendations for Special Populations

We found PEP recommendations for special populations in the guidelines of Bangladesh [23], Bhutan [24], Cambodia [25], China [26], India [27], Malaysia [30], Pakistan [31], Philippines [32], and Sri Lanka [33]. Chinese guidelines have an additional entry for PrEP recommendations in those populations [26].

PEP recommendations

- Pregnant and lactating women: The guidelines of Bangladesh, Bhutan, Cambodia, India, Pakistan, Philippines, and Sri Lanka state that there is no contraindication for vaccination in this population. The Chinese guidelines do not directly state whether PEP should be given or not, however they make reference to studies demonstrating that rabies vaccines are safe for pregnant women and for the fetus.
- Aged population and individuals with comorbidities: the same as above in the guidelines of Bangladesh, India, Pakistan, Philippines, and Sri Lanka.
- Immunocompromised population: full PEP and IM route is recommended in the guidelines of Bangladesh, Bhutan, India, Malaysia, Philippines, and Sri Lanka. There is no special reference to this population in the guidelines of Cambodia and Pakistan. Chinese guidelines indicate that passive immunization can be administered in patients with immunodeficiency disorder and that the antibody response should be closely monitored.
- Individuals on treatment for malaria taking chloroquine: ID is contraindicated and IM is recommended in the guidelines of Bangladesh, Cambodia, India, Malaysia, Pakistan, Philippines, and Sri Lanka.
- Other populations: Philippine’s guidelines also note that IM is contraindicated for individuals with hematologic conditions, for whom the ID route should be chosen and that ID is contraindicated for individuals with chronic liver disease.

*Chinese PrEP recommendations:* Only in the Chinese guidelines, we found recommendations specific to PrEP. According to these, PrEP can be delayed in case of (a) pregnancy; (b) acute febrile disease or other acute disease; (c) active chronic disease; (d) use of steroids and immunosuppressive products. PrEP is not recommended for patients with immunodeficiency disease.

## 4. Discussion

### 4.1. Characteristics of Human Rabies Burden

In endemic areas rabies disease is largely underreported, and reliable occurrence data are often scarce or non-existent [2,3,43]. Patients often seek no treatment [44], and some leave hospital against medical advice believing there is no cure [45] or because treatment cannot be offered [3]. Laboratory confirmation is sought in only a limited number of cases [44,46] because of limited resources and training [46,47]. Information systems for the collection of rabies cases may not be available [46] and often local authorities do not report rabies cases to central authorities [44,46]. In many countries, the officially reported numbers of rabies cases and deaths are substantially lower than the actual numbers [1]. In Cambodia for example, the incidence of human rabies deaths was estimated to be 5.8 per 100,000, which was 15 times higher than the officially reported incidence [48].

Younger children who are unable to protect themselves are at higher risk because they may interact with dogs in a manner perceived as threatening by the animal, e.g., stepping on the tail or trying to play when the dogs are eating [3,4,49,50]. Because of their smaller stature, children are more prone to get bitten multiple times on the face, head, and neck, being thus exposed to the more severe type of bites with the shortest incubation period [51,52]. Furthermore, children do no not always report minor bites or exposures by licks to their parents [52,53].

### 4.2. Rabies: An Unjustified Disease Burden

The development of vaccines against rabies started 100 years ago, and highly effective life-saving vaccines are currently available [19]. There is also a heightened awareness of rabies symptoms and inevitable fatal outcome without appropriate treatment [5,54,55]. Despite these advances, important knowledge gaps still exist. For example there are still those unaware that the wound must be immediately washed with soap and water [55,56], or that a laboratory test can confirm or reject suspected rabies in biting animal [55]. Furthermore, the rural population has limited access to vaccination centers as they are usually located in big cities, many animal bite victims do not seek medical care, and most infected individuals die at home [2,13]. In addition, RIG is often not made available because a large fraction of the population cannot afford it [56,57]. Poor people, living far from vaccination centers cannot afford to travel back-and-forth for injections, even if the vaccine is provided free of charge, and they often fail to complete complicated vaccination schedules [48,56]. The problem is further accentuated by a lack of access to vaccines and RIG globally.

### 4.3. Rabies Vaccination Schedules Across Asia: Current Situation

#### PEP Recommendations

Adequate wound cleaning can reduce the number of infectious viral particles inoculated via saliva during the bite from the rabid animal [58]. Improper or incomplete wound care is one of the reasons for PEP failure [59]. Thorough washing of the wound can eliminate or substantially reduce the viral load [60]. Unfortunately, this critical intervention is often undervalued or ignored [60]. The level of detail on wound washing techniques were highly heterogeneous in the national guidelines, suggesting a local need for specific operational guidelines on wound treatment.

Although international guidelines on the provision of RIG are clear, local recommendations are often different and almost universally the use of RIG in practice is not aligned to international recommendations due to significant access barriers [61].

The 5-dose Essen and 4-dose Zagreb IM regimens are considered equivalent in countries where both are recommended, except for the Bangladeshi guidelines that clearly favor the Zagreb regimen. The Zagreb regimen, however, is not endorsed by all national guidelines. 

ID PEP vaccination was endorsed in nine national guidelines; however, the value of ID administration in reducing costs was not considered regarding PEP in six national guidelines or other official documents. Clearly, there is still a need to emphasize the cost savings achieved using ID administration as opposed to the cost of the five IM regimen of similar vaccine efficacy. However, these cost savings can only occur in facilities with well-trained staff and with sufficient patients presenting with bite wounds to ensure that the maximum number of doses are extracted per vial of vaccine. With no preservative in the available vaccines, the vial should not be left open for longer than 6–8 h [1,13] and for smaller clinics this will not result in any cost saving vs. IM.

There are limited recommendations on the vaccination of patients who have a history of vaccination against rabies (PEP or PrEP). This could lead to overtreatment of patients presenting for treatment on multiple occasions. Given the risk factors for being bitten are associated with socio-economic factors, e.g., rural environments, working outdoors, it is likely that those who receive a bite are at a higher risk of subsequent bites than the general population. As such, multiple exposures are likely to occur. Clearer guidance could result in reduced use of vaccine and, where available, RIG for these patients.

Pregnancy, lactation, infancy, older age and comorbidities are not considered contraindications in the few guidelines that make reference to vaccination for special populations. Immunosuppressed individuals might have an inadequate antibody response in rabies PEP; however, specific recommendations can be found in very few guidelines.

### 4.4. PrEP Recommendations

Most country recommendations on patients for whom PrEP should be considered are aligned to WHO. Although recommendations are in place, uptake remains low because of complicated schedules, cost, and competing priorities especially within the context of limited PEP vaccine supply or resources for rabies control [62]. Less complicated vaccination schedules with shorter regimens and fewer doses would make PrEP simpler and reduce associated costs [62]. In addition, educational campaigns and rabies prevention and elimination programs should be conducted in areas where the infection rate is high; one such program was introduced in 2007 in the Bohol district of the Philippines, an area with the highest rabies incidence in the country [63]. The program included free routine PrEP for children aged 5–14 years and lasted four years (2007–2010) [52,62]. Up to April 2010, this program achieved high PrEP vaccination coverage (47%) of the target population [62].

Booster recommendations are not included in all guidelines and this may become an additional impediment to the optimal use of PrEP as there is little agreement on duration of protection or clinical benefit. Guidelines are more established for individuals at continual risk (lab workers, veterinarians, animal health workers) than for individuals at increased risk (children living in endemic areas, or travelers), and the intervals for serological follow-up (a test that is not consistently available everywhere) vary. It should also be noted that serological follow-up, even when recommended, is rarely practical or affordable. PrEP may be associated with cost savings because a previously vaccinated person needs shorter PEP and no RIG [62]. Children may benefit from receiving PrEP from 1 year of age and clear boosting recommendations are needed [62].

### 4.5. Country-Specific Information for Countries Whose National Guidelines Were Not Retrieved

In *Hong Kong* public hospitals, 10,255 individuals received PEP between 2000 and 2004, all of whom received rabies vaccine and 1% also received RIG [38]. The country’s rabies control program contains disease surveillance systems, laboratory diagnostic testing, PEP, and wound management [38].

For *Lao PDR*, the SEARS initiative (South-East Asia Rabies Strategy, 2013) indicates the rabies control program was not clearly defined, human resources were short, the surveillance system was inadequate, and laboratory confirmation was unavailable [64].

*Brunei* and *Singapore* are considered as free-of-rabies areas [43]. Singapore has been free from rabies since the 1950s and this was achieved through specific legislation, with which several rabies prevention strategies were implemented [43].

For *Myanmar*, the Association of Southeast Asian Nations 2016 report drew attention to the lack of a national rabies control strategy, low level of awareness and surveillance, and limited funding for rabies control [43].

In *Nepal*, public hospitals provide free post-exposure vaccination since 2007 [65]. RIG is available only in Kathmandu, and even there is mainly used by tourists and expats who can afford it [65].

### 4.6. Future Perspective

The Sustainable Development Goals (SDG), established by the September 2015 UN General Assembly, included the target to end epidemics of Neglected Tropical Diseases (NTDs) by 2030 [66]. Coinciding with these SDGs, WHO and OIE in collaboration with the FAO and supported by the GARC, have jointly set the goal for rabies elimination by 2030 [3,5]. Lessons learned from areas of the world where rabies has been successfully eliminated show that the “One Health” agenda will be the only way to achieve the 2030 goal of elimination of disease in Asia [5]. This framework resulted in substantial decrease in dog rabies incidence in 21 countries of Latin America and the Caribbean region, and several countries have been declared free of human rabies cases [67]. This was achieved thanks to a strong political commitment to control rabies in coordination with the Pan American Health Organization (PAHO). The program promoted mass canine vaccination, epidemiological surveillance, and provision of PEP and PrEP to people at risk. PEP became broadly available and, as part of the program, rabies vaccination centers were decentralized. PAHO’s Veterinary Public Health Program provided technical support for a disease notification system and coordinated actions between Ministries of Agriculture and Health and the executive councils of the WHO and OIE. Community education and involvement was part of the program to engage people in mass dog vaccination. The educational resources of the GARC were used to achieve this. Mass media campaigns on the radio, and in schools, health facilities, and similar organizations were staged to convince people to have their dogs vaccinated [67]. Similarly, in 2015, the Pan-African Rabies Control Network was launched, to integrate the One Health approach in the 33 member states, aiming to meet the 2030 target [68]. In a similar way, the Association of Southeast Asian Nations (ASEAN) developed the ASEAN Rabies Elimination Strategy in a One Health approach, integrating political, organizational, sociocultural, and technical collaboration to achieve rabies elimination by 2020 in the ASEAN member states, plus China, Japan, and Korea [43].

It is hoped that findings presented here might be useful to the authorities, health care providers, and patient organizations in the development and implementation of their initiatives for rabies elimination. This is more likely attainable through increased awareness of disease and encouraging the adoption of uniform rabies vaccination across Asia to ensure optimization of resource utilization, most often in resource-constrained environments. In that respect, the findings of the SAGE Working Group on rabies vaccines and rabies immunoglobulins will be highly significant.

To the best of our knowledge, this was the first work summarizing national rabies guidelines of the endemic Asian countries. However, we were unable to recover all national guidelines. Lack of information on vaccines’ origin and shortages are further limitations. Our work could not retrieve real-life data on proportions of use of ID and IM; a future prospective study would be necessary in that respect.

## 5. Conclusions

National recommendations across these countries differ and while some guidelines are closely aligned to the WHO recommendations, other countries specify PEP schedules that are very demanding on resources. A lot of progress has been made with respect to rabies control programs in many countries; however, efforts should be continued through closer collaboration between human and animal health sectors to meet the 2030 goal for rabies elimination. These efforts will have the opportunity to incorporate the most current findings of the SAGE Working Group on rabies vaccines and rabies immunoglobulins.

## Figures and Tables

**Table 1:** World Health Organization (WHO) recommendations for human rabies management.

Categories of Exposure to Suspected or Confirmed Rabid Animal and Actions Required	
**Category I**	Touching or feeding animals
	Licks on intact skin
	Contact of intact skin with secretions or excretions of a rabid animal or human case
	*Action required:* not regarded as exposures, no post-exposure prophylaxis is required
**Category II**	Nibbling of uncovered skin
	Minor scratches or abrasions without bleeding
	*Action required*: thorough local wound care and vaccine injection as soon as possible
**Category III**	Single or multiple transdermal bites or scratches
	Contamination of mucous membrane with saliva from licks
	Licks on broken skin
	Exposures to bat bites or scratches
	*Action required:* thorough local wound care and administration of vaccine and RIG as soon as possible
**Passive Immunization: Rabies Immunoglobulin (RIG)**	
**Target population**	All people with category III exposure, bites to the head, neck, face, and genitals
	Immunodeficient people with category II exposure
	Not for previously vaccinated individuals
**Type, dose**	Human rabies immunoglobulin: 20 IU/kg body weight
	Equine immunoglobulin: 40 IU/kg body weight
	F(ab’)_2_ products of equine immunoglobulin: 40 IU/kg body weight
**Time and site**	One administration as soon as possible, and within seven days from vaccination
	Into or around the wound site or sites. The remaining RIG (if any) should be administered IM at a site distant from the vaccination site
**Active Immunization: Vaccines**	
**Types**	Cell culture vaccine (CCV) and embryonated egg-based vaccines (CCEEVs):
	- Purified Vero cell rabies vaccine (PVRV)
	- Purified chick embryo cell vaccine (PCECV)
	- Human diploid cells vaccine (HDCV)
	and
	- Purified duck embryo vaccine (PDEV)
**Potency**	≥2.5 IU per single IM
**Route of administration, dose, vaccine type, and injection sites**	Intramuscular (IM), 1.0 mL or 0.5 mL (volume depending on the type of vaccine).
	- Vaccine type: any CCEEVs
	Sites:
	- Adults and children ≥2 years: in the deltoid area of the arm
	- Children <2 years: in the anterolateral area of thigh
	- Never in the gluteal area
	Intradermal (ID), 0.1 mL per ID site
	- Vaccine type: only PVRV or PCECV
	Sites:
	- The deltoids
	- Lateral thighs
	- Suprascapular areas
**Pre-Exposure Prophylaxis**	
**Indication**	Anyone at continual, frequent, or increased risk of exposure to the rabies virus, either as a result of their residence or occupation (laboratory staff, veterinarians, animal handlers and wildlife officers, etc.)
	Travelers with extensive outdoor exposure in rural high-risk areas
	Children living or visiting rabies-affected areas
**IM vaccination regimen**	3 doses, one IM dose on each of days 0, 7, and 21 or 28
**ID vaccination regimen**	3 doses, one ID injection of 0.1 mL on each of days 0, 7, and 21 or 28 *
**Booster injections**	Only for those working under continuous or frequent risk of exposure ^†^, if rabies-virus neutralizing antibody titers is <0.5 IU/mL
**Post-Exposure Prophylaxis**	
**Indication**	No vaccination is required for category I
	Immediate vaccination is required for category II
	Immediate vaccination is required for category III, (and administration of RIG)
**Wound care**	Immediate thorough washing and flushing, for 15 min, with soap or detergent
	Topical application of povidone iodine or other substances with virucidal activity
	A bleeding wound must be infiltrated with RIG
	Postpone suturing if possible; if suturing is necessary ensure that RIG has been applied locally
	Antibiotics and tetanus prophylaxis should be applied as appropriate for potentially contaminated wounds
**IM vaccination regimen ^§^**	*5-dose, Essen regimen (1-1-1-1-1):*
	one dose on each of days 0, 3, 7, 14, and 28
	*4-dose, Zagreb regimen (2-0-1-0-1 or 2-1-1):*
	2 doses on day 0 (one in each of the two deltoid or thigh sites) followed by one dose on each of days 7 and 21
	*4-dose shortened Essen regimen (1-1-1-1-0) for fully immunocompetent, exposed people who received wound care + high quality RIG + WHO prequalified rabies vaccine:*
	One dose on each of days 0, 3, 7, and 14
	*For immunocompromised individuals including patients with HIV/AIDS:*
	5-dose CCEEV regimen + wound care + local infiltration with human RIG. Evaluation of the rabies-virus neutralizing antibody 2–4 weeks after vaccination and administration of an additional vaccine dose if needed.
**ID vaccination regimen ^§^**	*The updated Thai Red Cross regimen (2-2-2-0-2):*
	Injections of 0.1 mL at two sites (deltoid and thigh) on each of days 0, 3, 7, and 28
**Short Post-Exposure Prophylaxis for Previously Vaccinated Individuals**	
*Exposed or re-exposed individuals, or individuals with rabies-virus neutralizing antibody titers of ≥0.5 IU/mL:*	
- One CCEEV IM dose OR one CCV ID dose on each of days 0 and 3, OR	
- One visit four site: four ID injections at a single visit	
- No RIG should be applied	
*Individuals exposed or re-exposed three months after complete vaccination:*	
- Wound care and booster vaccination if the dog or cat is healthy, vaccinated and available for an observation period of 10 days	
*Individuals with category III re- exposure who were vaccinated with a vaccine of unproven potency, or have received an incomplete course of vaccination:*	
- Full post-exposure vaccination course + RIG	

Sources: Rabies vaccines: WHO position paper (2010) [13] and WHO Expert Consultation on Rabies (2013) [1]; Abbreviations: CCEEV, cell culture vaccine and embryonated egg-based vaccines; CCV, cell culture vaccine; HDCV, human diploid cells vaccine; ID, intradermal; IM, intramuscular; PCECV, purified chick embryo cell vaccine; PDEV, purified duck embryo vaccine; PVRV, purified Vero rabies vaccine; RIG, rabies immunoglobulin; WHO, World Health Organization; * within 6–8 h, several individuals should be vaccinated in order to utilize all the volume of the opened vials, reducing thus the overall cost; ^†^ antibody monitoring, is preferred to booster injections. Every six months for those at risk of exposure to high concentrations of live rabies virus (e.g., laboratory workers dealing with rabies virus and other lyssaviruses), and every two years for those not at continuous risk of exposure (e.g., veterinarians and animal health officers); ^§^ at exceptional circumstances and when it is not possible to complete post-exposure prophylaxis with the same CCEEV, a rabies CCV fulfilling the WHO requirements should be used.

**Table 2:** Summary of available information on vaccination schedules, within the retrieved documents, by country.

Country	Document Title	Published by, (Year)	Available Vaccine Types (Route)	PEP Vaccination Schedule	PrEP Vaccination Schedule
**Bangladesh**	National Guideline for Rabies Prophylaxis and Intra-dermal Application of Cell Culture Rabies Vaccines [23]	Disease Control Unit, Ministry of Health & Family Welfare (2010)	PVRV (IM, ID) PCECV (IM, ID) ID administration is recommended as cost-effective and the technique is thoroughly described (not indicated for immunocompromised individuals)	**IM:** 4-dose Zagreb (Essen schedule described but not favored by the government)	**IM:** 3 doses, one injection on each of days 0, 7, and 21 or 28
				**ID:** Updated Thai Red Cross	**ID:** 3 doses, one injection on each of days 0, 7, and 21 or 28
				**Re-exposed previously vaccinated:** 2 doses, one injection (IM or ID) on each of days 0 and 3, if re-exposure was ≤5 years after full PEP + wound washing (RIG not necessary)	**Booster:** 1 dose when titers <0.5 IU/mL, for individuals working under risk for rabies exposure, monitoring Ab titers every 6 months
**Bhutan**	National Guideline for Management of Rabies [24]	Ministry of Health, Department of Public Health Zoonotic Disease Program (2014)	HDCV (IM) PVRV (IM, ID)	**IM:** 5-dose Essen	**IM:** 3 doses, one injection on each of days 0, 7, and 21 or 28
				**ID:** Updated Thai Red Cross	**ID:** 3 doses, one injection on each of days 0, 7, and 21 or 28
				**Re-exposed previously vaccinated:** 2 doses (IM or ID), each on days 0 and 3, for those who have documented previous full vaccination.	**Booster:** 1 site ID at 1 year and every 3 years to lab staff, veterinarians, animal handlers, dog catchers, wildlife workers. Regular (exact timing not specified) monitoring of Ab titers and administration of a booster dose when titers <0.5 IU/mL.
**Cambodia**	Rabies Vaccine Procedure SOP-OPD-02-004 [25]	National Institute of Public Health, National Public Health Laboratory (2012)	PDEV (IM) PCECV (IM, ID) HDCV (IM) PVRV (IM, ID)	**IM:** 5-dose Essen 4-dose Zagreb	**IM:** 3 doses, one injection on each of days 0, 7, 21–28
				**ID:** The updated Thai Red Cross 8-site Oxford (8-0-4-0-1-1 injection sites on days 0, 7, 28 and 90)	**ID:** 3 doses, one injection on each of days 0, 7, 21–28
				**Re-exposed previously vaccinated:** 2 doses, one injection on each of days 0 and 3, in individuals who had receive at least 3 doses of any PEP regimen	
**China**	Technical Guidelines for Human Rabies Control and Prevention [26]	Chinese Center for Disease Control and Prevention (2016)	PVRV, (IM) HDCV, (IM) PHKCV, (IM) PCECV, (IM)	**IM:** 5-dose Essen 4-dose Zagreb	**IM:** 3 doses, one injection on each of days 0, 7, and 21 or 28
				**Re-exposed previously vaccinated:** 2 injections each on days 0 and 3 if >3 months have passed from previous full vaccination, if <3 months have passed, booster may be deferred if animal is healthy, vaccinated and accessible for observation	**Booster:** 1 booster dose when titers <0.5 IU/mL, for individuals working under risk for rabies exposure (monitoring every 6 months for lab workers and every 24 months for veterinarians/animal health officers)
**Hong Kong**	Web page: Rabies [37]	Center for Health Protection, Department of Health (2017)	(not specified)	Thorough wound cleansing Vaccination if necessary	Travelers to endemic areas
	Web page: Vaccine and Prophylaxis—Rabies Vaccination [39]	Travel Health Service, Department of Health (2012)	(not specified)	(not specified)	For Travelers to endemic areas, one month before the trip: 3 doses, one injection on each of days 0, 7, and 21 or 28 (Route is not specified)
	Scientific Committee on Emerging and Zoonotic Diseases — Prevention and Control of Rabies [38]	Center for Health Protection (2005)	(not specified)	(not specified)	(not specified)
**India**	National Guidelines on Rabies Prophylaxis [27]	National Center for Disease Control (2015)	PCECV (IM, ID) PVRV (IM, ID) HDCV (IM) PDEV (IM)	**IM:** 5-dose Essen	**IM:** 3 doses, one injection on each of days 0, 7, and 21 or 28
				**ID:** Updated Thai Red Cross	**Booster:** 1 booster dose when Ab titers <0.5 IU/mL, for individuals working under risk for rabies exposure (monitoring every 6 months for the first 2 years and every 24 months thereafter)
				**Re-exposed previously vaccinated:** 2 doses (IM or ID), each on days 0 and 3, for those who have received full vaccination and re-exposed	
	Indian Academy of Pediatrics (IAP) Recommended Immunization for Children Aged 0 through 18 years—India, 2016 and Updates on Immunization [28]	Indian Pediatrics (2016)	HDCV (IM) PCECV (IM) PDEV (IM) PVRV (IM) ID not in individual practice	**IM:** 5 doses, one dose on each day 0, 3, 7, 14, and 30, and for individuals with severe debility or immunosuppressed, and optional 6th dose on day 90	**IM:** 3 doses, one injection on each of days 0, 7, 21, or 28 (day 28 is preferred)
				**Re-exposed previously vaccinated:** 2 doses for those who have received full vaccination and re-exposed	
**Indonesia**	National Guidelines for Rabies Vaccination [29]	Center for Disease Control, Ministry of Health (2011)	PVRV (IM) PCECV (IM)	**IM:** 4-dose Zagreb	**IM:** 3 doses, one injection on each of days 0, 7, 21, or 28
				**Re-exposed previously vaccinated:** 1 dose if re-exposure occurred 3–12 months from full vaccination, no vaccination below 3 months, full vaccination over 12 months	
**Japan**	Questions and answers on rabies [40]	Ministry of Health (2017)	(not specified) ID is not approved RIG is not approved	**SC:** 6 doses on days 0, 3, 7, 14, 30, and 90	**SC:** week 0, 4 and month 6–12
				**Re-exposed previously vaccinated:** 2 injections for those who have received full vaccination, on days 0 and 3	
**Lao People’s Democratic Republic**	Intradermal application of rabies vaccines. Report of a WHO consultation. Bangkok, Thailand 2000 [41]	WHO, Communicable Disease Surveillance and Control (2000)	PVRV (IM) PCECV (IM) ID not yet used, RIG is rarely used due high cost The ID route is not used	(not specified)	(not specified)
**Malaysia**	Interim guideline for human rabies prevention & control [30]	Ministry of Health, Disease Control Division (2015)	PVRV (IM)	**IM:** 4-dose shortened Essen	(not specified)
				**Re-exposed previously vaccinated:** 2 doses on days 0 and 3	
**Pakistan**	Country Guidelines for Prevention of Rabies. Pakistan Rabies Control Programme [31]	WHO in consultation with Provincial Health Departments (2013)	PVRV (IM, ID) PCECV (IM, ID) PDEV (IM)	**IM:** 4-dose Zagreb	**IM:** 3 doses, one injection on each of days 0, 7, 21, or 28
				**ID:** The updated Thai Red Cross	**ID:** 3 doses, one injection on each of days 0, 7, 21, or 28
				**Re-exposed previously vaccinated:** 2 injections (IM or ID), for those who have received full vaccination, on days 0 and 3	
**Philippines**	New Guidelines on the Management of Rabies Exposure [32]	Republic of Philippines. Department of Health (2014)	PVRV (IM, ID) PCECV (IM, ID) Among the first countries that adopted ID	**IM:** 5-dose Essen 4-dose Zagreb 4-dose shortened Essen	**IM:** 3 doses, one injection on each of days 0, 7, 21, or 28
				**ID:** The updated Thai Red Cross	**ID:** 3 doses, one injection on each of days 0, 7, 21, or 28
				**Re-exposed previously vaccinated:** 2 injections (IM or ID), each on days 0 and 3 if the re-exposed individual had previously receive full vaccination, and full vaccination if he/she had not receive full vaccination course	**Booster:** Routine booster for individual with occupational risk: 1 dose after one year from full vaccination and one more thereafter in case of Ab titers <0.5 IU/mL
**Republic of Korea**	Recommended Adult Immunizations for Foreign Travel [42]	Korea Society of Infectious Disease (2012)	(not specified)		3 doses
**Sri Lanka**	Protocol for anti-rabies post exposure therapy [33]	Director General of Health Services (2016)	PVRV (IM, ID) PCECV (IM, ID)	**IM:** 5-dose Essen 4-dose Zagreb	**IM:** 3 doses, one injection on each of days 0, 7, 21, or 28
				**ID:** Updated Thai Red Cross Modified 4-site ( 4-2-2-0-2): one dose at 4 sites on day 0 and one dose at 2 sites on days 3, 7, and 30	**ID:** 3 doses, one injection on each of days 0, 7, 21, or 28
				**Re-exposed previously vaccinated:** 2 injections (IM or ID), one on each of day 0 and 3, OR 4 ID at four sites on the same visit	**Booster:** One injection on the 1st year following full vaccination, and one more every five years
**Taiwan**	Post-exposure guidelines [34]	Centers for Disease Control (2016)	(not specified, ID not recommended)	**IM:** 5-dose Essen	**IM:** one dose, on day 0, 7, 21 or 28 (3 doses)
					**Booster:** For individuals at high risk (e.g., laboratory workers): 2 injections, one on each day 0 and 4 when Ab titers <0.5 IU/mL, For workers in animal health: One booster dose one year following full vaccination and every 3–5 years thereafter
**Thailand**	Thai rabies management guidelines [35]	Queen Saovabha Memorial Institute (2015)	PCEVC (IM, ID) PVRV (IM, ID) PDEV (IM)	**IM:** 5-dose Essen	**IM:** 3 doses, one injection on each of days 0, 7, 21, or 28
				**ID:** Updated Thai Red Cross	**ID:** 3 doses, one injection on each of days 0, 7, 21, or 28 Only for PVRV: 2 doses, one injection on each of days 0 and 28
				**Re-exposed previously vaccinated:** (a) 2 injections, each on day 0 and 3 if exposure occurred within 6 months from vaccination, no RIG; (b) same as previous or 4-site ID on a single visit if >6 months have elapsed from previous vaccination to exposure	**Booster:** For individuals at continuous or frequent risk of exposure: One booster vaccination if Ab titers <0.5 IU/mL
**Vietnam**	Guidelines on human rabies surveillance and prevention [36]	Ministry of Health (2014)	Cell culture vaccines (not specified)	**IM:** 5-dose Essen	**IM:** 3 doses, one injection on each of days 0, 7, 21, or 28 One repeated injection every year
				**ID:** Updated Thai Red Cross	**Booster:** One injection on 1st year from previous full vaccination and every year thereafter
				**Re-exposed previously vaccinated:** (a) 2 injections, each on days 0 and 3 if time from previous full vaccination <5 years; (b) Full vaccination if previous was not completed or >5 years have elapsed from previous full vaccination	

Abbreviations: Ab, antibody; APCRI, Association for Prevention and Control of Rabies in India; HDCV, human diploid cells vaccine; IAP, Indian Academy of Pediatrics; ID, intradermal; IM, intramuscular; PCECV, primary chick embryo cell vaccine; PDEV, purified duck embryo vaccine; PEP, post-exposure prophylaxis; PHKCV, primary hamster kidney cell vaccine; PrEP, pre-exposure prophylaxis; PVRV, purified Vero rabies cell vaccine; SC, subcutaneous.

**Table 3:** National guidelines endorsing the WHO recommended PEP vaccination regimens: Essen, Zagreb, and the updated Thai Red Cross.

Country	5-Dose Essen (1-1-1-1-1)	4-Dose Zagreb (2-0-1-0-1 or 2-1-1)	4-Dose Essen (1-1-1-1-0)	Updated Thai Red Cross (2-2-2-0-2)
Bangladesh	X	X		X
Bhutan	X			X
Cambodia	X	X		X
China	X	X		
India	X			X
Indonesia		X		
Malaysia			X	
Pakistan		X		X
Philippines	X	X	X	X
Sri Lanka	X	X		X
Taiwan	X			
Thailand	X			X
Vietnam	X			X

## Abbreviations

Ab	antibody
APCRI	Association for Prevention and Control of Rabies in India
CCEEV	cell culture vaccine and embryonated egg-based vaccines
CCV	cell culture vaccine
ERIG	equine immunoglobulin
FAO	Food and Agriculture Organization
GARC	Global Alliance for Rabies Control
HDCV	human diploid cells vaccine
HIV	human immunodeficiency virus
HRIG	human immunoglobulin
IAP	Indian Academy of Pediatrics
ID	intradermal
IM	intramuscular
NTD	Neglected Tropical Diseases
OIE	World Organization for Animal Health
PAHO	Pan American Health Organization
PCECV	purified chick embryo cell vaccine
PDEV	purified duck embryo vaccine
PEP	post-exposure prophylaxis
PHKCV	primary hamster kidney cell vaccine
PrEP	pre-exposure prophylaxis
PVRV	purified Vero rabies vaccine
RIG	rabies immunoglobulin
RVNA	rabies virus neutralizing antibodies
SAGE	Strategic Advisory Group of Experts
SC	subcutaneous
SDG	Sustainable Development Goals
SEARS	South-East Asia Rabies Strategy
UN	United Nations
WHO	World Health Organization

## References

[1] World Health Organization (2013). WHO Expert Consultation on Rabies (Second Report).

[2] Hampson K., Coudeville L., Lembo T., Sambo M., Kieffer A., Attlan M., Barrat J., Blanton J.D., Briggs D.J., Cleaveland S. (2015). Estimating the global burden of endemic canine rabies. PLoS Negl. Trop. Dis..

[3] Fahrion A., Mikhailov A., Abela-Ridder B., Giacinti J., Harries J. (2016). Human Rabies Transmitted by Dogs: Current Status of Global Data, 2015.

[4] Food and Agriculture Organization of the United Nations (FAO) (2013). Developing a Stepwise Approach for Rabies Prevention and Control.

[5] World Health Organization, World Organisation for Animal Health (OIE), Food and Agriculture Organization of the United Nations (FAO), Global Alliance for Rabies Control (GARC) (2015). Global Elimination of Dog-Mediated Human Rabies: The Time is Now.

[6] Lavan R.P., King A.I., Sutton D.J., Tunceli K. (2017). Rationale and support for a One Health program for canine vaccination as the most cost-effective means of controlling zoonotic rabies in endemic settings. Vaccine.

[7] Cleaveland S., Lankester F., Townsend S., Lembo T., Hampson K. (2014). Rabies control and elimination: A test case for One Health. Vet. Rec..

[8] Wangdi K., Ward M.P. (2012). Human and animal rabies prevention and control cost in Bhutan, 2001–2008: The cost-benefit of dog rabies elimination. Vaccine.

[9] Maurya I., Vagholkar K., Patel B., Siddiqui M., Tiwari S., Maurya P. (2015). State of globe: Rabies: The lethality since antiquity!. J. Glob. Infect. Dis..

[10] Canine Rabies Blueprint The Stepwise Approach towards Rabies Elimination: A Tool for Planning and Evaluation. http://caninerabiesblueprint.org/IMG/pdf/sare_outline_2017_f.pdf.

[11] Sugiyama M., Ito N. (2007). Control of rabies: Epidemiology of rabies in Asia and development of new-generation vaccines for rabies. Comp. Immunol. Microbiol. Infect. Dis..

[12] Dodet B., Goswami A., Gunasekera A., de Guzman F., Jamali S., Montalban C., Purba W., Quiambao B., Salahuddin N., Sampath G., Tang Q., Tantawichien T., Wimalaratne O., Ziauddin A. (2008). Rabies awareness in eight Asian countries. Vaccine.

[13] World Health Organization (2010). Rabies Vaccines: WHO Position Paper.

[14] Wilde H. (2007). Failures of post-exposure rabies prophylaxis. Vaccine.

[15] Madhusudana S.N., Mani R.S. (2014). Intradermal vaccination for rabies prophylaxis: Conceptualization, evolution, present status and future. Expert Rev. Vaccines.

[16] Narayana A., Manoharan A., Narayan M.S., Kalappa S.M., Biligumba G., Haradanahalli R., Anand A.M. (2015). Comparison of safety and immunogenicity of 2 WHO prequalified rabies vaccines administered by one week, 4 site intradermal regimen (4-4-4-0-0) in animal bite cases. Hum. Vaccines Immunother..

[17] Tarantola A., Blanchi S., Cappelle J., Ly S., Chan M., In S., Peng Y., Hing C., Taing C.N., Ly S. (2017). Rabies postexposure prophylaxis (PEP) noncompletion after dog bites: Estimating the unseen to meet the needs of the underserved. Am. J. Epidemiol..

[18] Dhaduk K.M., Unadkat S.V., Katharotiya P.R., Mer A.R., Chaudhary M.C., Prajapati M.M. (2016). Case profile, volume analysis, and dropout rate of antirabies vaccination regimens among animal bite victims in Gujarat. Indian J. Public Health.

[19] Tarantola A. (2017). Four thousand years of concepts relating to rabies in animals and humans, its prevention and its cure. Trop. Med. Infect. Dis..

[20] Mahendra B.J., Narayana D.A., Agarkhedkar S., Ravish H.S., Harish B.R., Agarkhedkar S., Madhusudana S.N., Belludi A., Ahmed K., Jonnalagedda R. (2015). Comparative study on the immunogenicity and safety of a purified chick embryo cell rabies vaccine (PCECV) administered according to two different simulated post exposure intramuscular regimens (Zagreb versus Essen). Hum. Vaccines Immunother..

[21] World Health Organization Strategic Advisory Group of Experts (SAGE) Working Group on Rabies Vaccines and Rabies Immunoglobulins (Established July 2016). http://www.who.int/immunization/policy/sage/sage_wg_rabies_jul2016/en/.

[22] World Health Organization (2017). Human Rabies: 2016 Updates and Call for Data.

[23] Ahmad Z., Amin R., Ussaman S., Jamil M., Ahmed M., Bangladesh Disease Control Unit Directorate General of Health Services (2010). National Guideline for Rabies Prophylaxis and Intra-Dermal Application of Cell Culture Rabies Vaccines.

[24] Royal Government of Bhutan Ministry of Health Department of Pubic Health Zoonotic Disease Program (2014). National Guideline for Management of Rabies.

[25] Vanneth D. (2012). Rabies Vaccine Procedure SOP-OPD-02–004, Rev. 2.

[26] Zhou H., Li Y., Chen R.F., Tao X.Y., Yu P.C., Cao S.C., Li L., Chen Z.H., Zhu W.Y., Yin W.W. (2016). Technical guideline for human rabies prevention and control (2016). Zhonghua Liu Xing Bing Xue Za Zhi.

[27] Indian National Centre for Disease Control (2015). National Rabies Control Programme. National Guidelines on Rabies Prophylaxis.

[28] Vashishtha V.M., Choudhary J., Jog P., Yadav S., Unni J.C., Kamath S., Sachdeva A., Srirampur S., Prajapati B., Parekh B. (2016). Indian Academy of Pediatrics (IAP) recommended immunization schedule for children aged 0 through 18 years—India, 2016 and updates on immunization. Indian Pediatr..

[29] Indonesia Kementerian Kesehatan RI Directorat Jenderal PP dan PL (2011). Pedoman Pelaksanaan Program Penanggulangan Rabies Di Indonesia.

[30] Malaysia Disease Control Division, Ministry of Health (2015). Interim Guideline for Human Rabies Prevention & Control in Malaysia.

[31] Pakistan Rabies Control Programme (2013). Country Guidelines for the Prevention of Rabies.

[32] Republic of the Philippines (2014). New Guidelines on the Management of Rabies Exposures.

[33] Mahipala P.G., Director General of Health Services (2016). Protocol for Anti-Rabies Post Exposure Therapy (PET).

[34] Taiwan Center for Disease Control (2016) Rabies Post Exposure Guidelines. Dissertation. http://www.cdc.gov.tw/professional/page.aspx?treeid=beac9c103df952c4&nowtreeid=b2db963d0bad6639.

[35] Thai Department of Disease Control MOPH (2015). Rabies Guideline and Frequent Questions.

[36] Socialist Republic of Vietnam, Ministry of Health (2014). Guidelines on Human Rabies Surveillance and Prevention.

[37] Hong Kong Center for Health Protection, Department of Health The Goverment of Hong Kong Special Administrative Region Rabies. http://www.chp.gov.hk/en/content/9/24/3149.html.

[38] Hong Kong Center for Health Protection (2005). Scientific Committee on Emerging and Zoonotic Diseases—Prevention and Control of Rabies.

[39] Hong Kong Travel Health Service Department of Health The Government of Hong Kong Special Administrative Region Vaccine and Prophylaxis—Rabies Vaccination. http://www.travelhealth.gov.hk/english/vaccine_prophylaxis/rabies.html.

[40] Japan Ministry of Health Labour and Welfare Q & A on Rabies Vaccination Guidelines. http://www.mhlw.go.jp/bunya/kenkou/kekkaku-kansenshou10/07.html.

[41] World Health Organization (2000). Intradermal Application of Rabies Vaccines—Report of a WHO Consultation, Bangkok, Thailand.

[42] Korea Society of Infectious Disease Recommended Adult Immunization for Foreign Travel. http://www.ksid.or.kr/file/vaccine_eng.pdf.

[43] Association of Southeast Asian Nations (ASEAN) (2016). ASEAN Rabies Elimination Strategy.

[44] Knobel D.L., Cleaveland S., Coleman P.G., Fevre E.M., Meltzer M.I., Miranda M.E., Shaw A., Zinsstag J., Meslin F.X. (2005). Re-evaluating the burden of rabies in Africa and Asia. Bull. World Health Organ..

[45] Sudarshan M.K., Madhusudana S.N., Mahendra B.J., Rao N.S., Narayana D.A., Rahman S.A., Meslin F.X., Lobo D., Ravikumar K., Gangaboraiah (2007). Assessing the burden of human rabies in India: Results of a national multi-center epidemiological survey. Int. J. Infect. Dis..

[46] Taylor L.H., Hampson K., Fahrion A., Abela-Ridder B., Nel L.H. (2017). Difficulties in estimating the human burden of canine rabies. Acta Trop..

[47] Banyard A.C., Horton D.L., Freuling C., Muller T., Fooks A.R. (2013). Control and prevention of canine rabies: The need for building laboratory-based surveillance capacity. Antivir. Res..

[48] Ly S., Buchy P., Heng N.Y., Ong S., Chhor N., Bourhy H., Vong S. (2009). Rabies situation in Cambodia. PLoS Negl. Trop. Dis..

[49] Daniels D.M., Ritzi R.B., O’Neil J., Scherer L.R. (2009). Analysis of nonfatal dog bites in children. J. Trauma Acute Care Surg..

[50] Dhand N.K., Gyeltshen T., Firestone S., Zangmo C., Dema C., Gyeltshen R., Ward M.P. (2011). Dog bites in humans and estimating human rabies mortality in rabies endemic areas of Bhutan. PLoS Negl. Trop. Dis..

[51] Schalamon J., Ainoedhofer H., Singer G., Petnehazy T., Mayr J., Kiss K., Hollwarth M.E. (2006). Analysis of dog bites in children who are younger than 17 years. Pediatrics.

[52] Dodet B. (2010). Report of the sixth AREB meeting, Manila, The Philippines, 10–12 November 2009. Vaccine.

[53] Sudarshan M.K., Parthasarathy A., Kundu R., Agrawal R. (2013). Guidelines for Rabies Prophylaxis in Children. Textbook of Pediatric Infectious Diseases.

[54] Matibag G.C., Kamigaki T., Kumarasiri P.V., Wijewardana T.G., Kalupahana A.W., Dissanayake D.R., De Silva D.D., Gunawardena G.S., Obayashi Y., Kanda K. (2007). Knowledge, attitudes, and practices survey of rabies in a community in Sri Lanka. Environ. Health Prev. Med..

[55] Dhand N.K., Rai B.D., Tenzin S., Tsheten K., Ugyen P., Singye K., Ward M.P. (2012). Community-based study on knowledge, attitudes and perception of rabies in Gelephu, south-central Bhutan. Int. Health.

[56] Salahuddin N., Gohar M.A., Baig-Ansari N. (2016). Reducing cost of rabies post-exposure prophylaxis: experience of a tertiary care hospital in Pakistan. PLoS Negl. Trop. Dis..

[57] Hampson K., Cleaveland S., Briggs D. (2011). Evaluation of cost-effective strategies for rabies post-exposure vaccination in low-income countries. PLoS Negl. Trop. Dis..

[58] Hemachudha T., Ugolini G., Wacharapluesadee S., Sungkarat W., Shuangshoti S., Laothamatas J. (2013). Human rabies: Neuropathogenesis, diagnosis, and management. Lancet Neurol..

[59] Rupprecht C.E., Briggs D., Brown C.M., Franka R., Katz S.L., Kerr H.D., Lett S., Levis R., Meltzer M.I., Schaffner W. (2009). Evidence for a 4-dose vaccine schedule for human rabies post-exposure prophylaxis in previously non-vaccinated individuals. Vaccine.

[60] Dodet B. (2007). An important date in rabies history. Vaccine.

[61] Tarantola A., Ly S., In S., Ong S., Peng Y., Heng N., Buchy P. (2015). Rabies vaccine and rabies immunoglobulin in Cambodia: use and obstacles to use. J. Travel Med..

[62] Kessels J.A., Recuenco S., Navarro-Vela A.M., Deray R., Vigilato M., Ertl H., Durrheim D., Rees H., Nel L.H., Abela-Ridder B. (2017). Pre-exposure rabies prophylaxis: A systematic review. Bull. World Health Organ..

[63] Davlin S.L., Lapiz S.M., Miranda M.E., Murray K.O. (2014). Knowledge, attitudes, and practices regarding rabies in Filipinos following implementation of the Bohol Rabies Prevention and Elimination Programme. Epidemiol. Infect..

[64] The South-East Asia Dog Rabies Elimination Strategy (SEARS) (2013). Rabies.

[65] Devleesschauwer B., Aryal A., Sharma B.K., Ale A., Declercq A., Depraz S., Gaire T.N., Gongal G., Karki S., Pandey B.D. (2016). Epidemiology, impact and control of rabies in Nepal: A systematic review. PLoS Negl. Trop. Dis..

[66] GBD 2015 SDG Collaborators (2016). Measuring the health-related Sustainable Development Goals in 188 countries: A baseline analysis from the Global Burden of Disease Study 2015. Lancet.

[67] Vigilato M.A., Clavijo A., Knobl T., Silva H.M., Cosivi O., Schneider M.C., Leanes L.F., Belotto A.J., Espinal M.A. (2013). Progress towards eliminating canine rabies: Policies and perspectives from Latin America and the Caribbean. Philos. Trans. R. Soc. Lond. B Biol. Sci..

[68] Scott T.P., Coetzer A., de B.K., Wright N., Nel L.H. (2015). The Pan-African Rabies Control Network (PARACON): A unified approach to eliminating canine rabies in Africa. Antivir. Res..

